# A Long QT Mutation Substitutes Cholesterol for Phosphatidylinositol-4,5-Bisphosphate in KCNQ1 Channel Regulation

**DOI:** 10.1371/journal.pone.0093255

**Published:** 2014-03-28

**Authors:** Fabien C. Coyan, Fayal Abderemane-Ali, Mohamed Yassine Amarouch, Julien Piron, Jérôme Mordel, Céline S. Nicolas, Marja Steenman, Jean Mérot, Céline Marionneau, Annick Thomas, Robert Brasseur, Isabelle Baró, Gildas Loussouarn

**Affiliations:** 1 l'institut du thorax, Institut National de la Santé et de la Recherche Médicale, Nantes, France; 2 Unité Mixte de Recherche 6291, Centre National de la Recherche Scientifique, Nantes, France; 3 Unité de Formation et de Recherche de Médecine, Université de Nantes, Nantes, France; 4 Institut de Pharmacologie et de Biologie Structurale, Centre National de la Recherche Scientifique, Toulouse, France; 5 Centre de Biophysique Moléculaire Numérique, University of Liège, Gembloux, Belgium; Indiana University School of Medicine, United States of America

## Abstract

**Introduction:**

Phosphatidylinositol-4,5-bisphosphate (PIP_2_) is a cofactor necessary for the activity of KCNQ1 channels. Some Long QT mutations of KCNQ1, including R243H, R539W and R555C have been shown to decrease KCNQ1 interaction with PIP_2_. A previous study suggested that R539W is paradoxically less sensitive to intracellular magnesium inhibition than the WT channel, despite a decreased interaction with PIP_2_. In the present study, we confirm this peculiar behavior of R539W and suggest a molecular mechanism underlying it.

**Methods and Results:**

COS-7 cells were transfected with WT or mutated KCNE1-KCNQ1 channel, and patch-clamp recordings were performed in giant-patch, permeabilized-patch or ruptured-patch configuration. Similar to other channels with a decreased PIP_2_ affinity, we observed that the R243H and R555C mutations lead to an accelerated current rundown when membrane PIP_2_ levels are decreasing. As opposed to R243H and R555C mutants, R539W is not more but rather less sensitive to PIP_2_ decrease than the WT channel. A molecular model of a fragment of the KCNQ1 C-terminus and the membrane bilayer suggested that a potential novel interaction of R539W with cholesterol stabilizes the channel opening and hence prevents rundown upon PIP_2_ depletion. We then carried out the same rundown experiments under cholesterol depletion and observed an accelerated R539W rundown that is consistent with this model.

**Conclusions:**

We show for the first time that a mutation may shift the channel interaction with PIP_2_ to a preference for cholesterol. This *de novo* interaction wanes the sensitivity to PIP_2_ variations, showing that a mutated channel with a decreased affinity to PIP_2_ could paradoxically present a slowed current rundown compared to the WT channel. This suggests that caution is required when using measurements of current rundown as an indicator to compare WT and mutant channel PIP_2_ sensitivity.

## Introduction

Cholesterol regulates several ion channels, and changes in membrane cholesterol levels provoke various effects depending on the channel type (reviewed in [1], [2]). Regarding inwardly rectifying K^+^ channels (Kir), cholesterol effects have been studied in detail. Effects of cholesterol increase or depletion vary among families and even among channels of the same family. Similarly to Kir channels, functional effects of cholesterol on voltage-gated (Kv) channel activity are highly variable: an increase in Kv2.1 current in Drosophila neurons [3] and a suppression of the same current in mammalian pancreatic β-cells [4]. Adding more complexity, effects on the cardiac potassium channel KCNQ1 are variable depending on the drug used to decrease cholesterol levels [5], [6]. More specifically, Probucol - known as a cholesterol depleting agent - is able to decrease the coexpressed KCNE1/KCNQ1 current amplitude [5] without decreasing cholesterol levels in CHO-K1 [6]. Simvastatin and triparanol are more specific since their main effects (activation kinetics) are similar and correlated to a cholesterol decrease. Clearly, cholesterol effects on KCNQ1 channels remain to be studied in more detail.

Phosphatidylinositol-4,5-bisphosphate (PIP_2_) is abundant in cholesterol-rich membrane domains [7]. The mechanisms by which PIP_2_ regulates several channels, including KCNQ1, have been studied in much greater detail than the mechanism of their regulation by cholesterol [8]–[16]. PIP_2_ is a minor acidic membrane lipid found primarily in the inner leaflet of the plasma membrane. It has been shown to be a necessary cofactor for a wide variety of ion channels and transporters [17]. In Kir and Kv channels, several stimuli impact channel activity by decreasing available PIP_2_ or modulating channel-PIP_2_ interactions [8], [9], [18]–[20]. Consistent with PIP_2_ regulating channel activities, mutations that impair channel-PIP_2_ interactions play a crucial role in channelopathies. Regarding KCNQ1, we have shown that type 1 long QT (LQT1) syndrome can be associated with a decrease in KCNQ1-PIP_2_ interactions provoked by mutations in the S4–S5 linker (R243H) and in the C-terminal domain CTD (R539W and R555C) [10]. This has since been confirmed for mutations R539W and R555C [11], [12].

In previous studies, PIP_2_ sensitivity of the KCNQ1 mutants R243H, R539W and R555C was determined by using the soluble short acyl chains analog diC8-PIP_2_ [10]. Since then, PIP_2_-regulated Kv11.1 channels have been shown to be insensitive to diC8-PIP_2_ [21], suggesting that channel affinity for PIP_2_ and diC8-PIP_2_ might be quite different. Moreover, the R539W behavior seems paradoxical since, in COS-7 cells, this mutant was shown to be less sensitive than WT to intracellular Mg^2+^, which decreases PIP_2_ availability through masking its negative charges [10]. In general, any mutation that reduces channel affinity to PIP_2_ provokes an increased inhibition by Mg^2+^ [18]. Hence, we set out to use other approaches to further study the PIP_2_ affinity of KCNQ1 mutant channels in the presence of KCNE1. We measured the kinetics of Mg^2+^-, wortmannin- and Ci-VSP-induced current rundown and confirmed a decreased PIP_2_ affinity for two of the three mutants. To our surprise, for the third mutant (R539W) the current is running down more slowly when available PIP_2_ is decreased. We therefore hypothesized that the KCNQ1-R539W mutation leads to the stabilization of an open-pore conformation shortcutting the PIP_2_ effect on the concerted opening. Using both modeling and functional analyses, we show for the first time that a mutation in the CTD domain shifts the channel interaction with PIP_2_ to a preference for cholesterol.

In conclusion, our study shows that cholesterol affects the rundown of R539W but not of WT KCNE1-KCNQ1 channels, suggesting that cholesterol specifically stabilizes opening of R539W channels and decreases their need for PIP_2_ to be open. Estimating a channel affinity to PIP_2_ from the kinetics of the current rundown when PIP_2_ decreases is an approach that has been commonly used. This study indicates that such an approach is not always relevant, since the mutation that disrupts the interaction with PIP_2_ may stabilize the channel open state through another mechanism.

## Methods

### Cell culture and transfection

The African green monkey kidney-derived cell line COS-7 was obtained from the American Type Culture Collection (Rockville, MD, USA) and cultured in Dulbecco's modified Eagle's medium (GIBCO, Paisley, Scotland). Cells were transfected with plasmids (2 µg per 35 mm dish) complexed with Fugene-6 (Roche Molecular Biochemical) for giant-patch and ruptured-patch experiments, or Jet-PEI (Polyplus-Transfection) for permeabilized-patch experiments, according to the standard protocol recommended by the manufacturers. We used a construct of hKCNQ1 fused to hKCNE1 (KCNE1-KCNQ1) to prevent variability caused by a variable KCNE1/KCNQ1 expression ratio [10]. This type of construct is justified by the fact that the cardiac channel is generated by the assembly of 4 KCNQ1 and up to 4 KCNE1 subunits [22]. For giant-patch experiments, the relative DNA composition was 80% pCDNA3.1 plasmid containing the human WT or mutated KCNE1-KCNQ1 concatemer [10], [23] and 20% pEGFP coding for the green fluorescent protein (Clontech). For permeabilized-patch experiments testing the effects of osmolarity, relative DNA composition was 40% pCDNA3.1 KCNE1-KCNQ1 and 60% pEGFP. For permeabilized-patch experiments testing the PKA-dependent channel upregulation, relative DNA composition was 20% pCDNA3.1-KCNE1-KCNQ1, 40% pEGFP and 40% pCDNA3-yotiao (a kind gift of Dr Robert S. Kass, Department of Pharmacology, College of Physicians & Surgeons, Columbia University, New York, NY, USA). For ruptured-patch experiments testing the effects of Ci-VSP, relative DNA composition was 20% pCDNA3.1-KCNE1-KCNQ1, 70% pEGFP and 10% pRFP-C1-Ci-VSP (a kind gift of Dr Dominik Oliver, Institute of Physiology and Pathophysiology, Philipps-Universität Marburg, Marburg, Germany).

### Electrophysiology

Forty-eight to seventy-two hours post-transfection, COS-7 cells were mounted on the stage of an inverted microscope and constantly perfused at a rate of 2 mL/min. Experiments were performed at room temperature for giant-patch and ruptured-patch configurations, or 35.0±1.0°C for permeabilized-patch configuration. Stimulation, data recording, and analysis were performed either by Acquis1 (Bio-logic Science Instruments, Claix, France) through an analog-to-digital converter (Tecmar TM100 Labmaster; Scientific Solution, Solon, OH, USA), or by pClamp10.4 through an analog-to-digital converter (Axon Digidata 1440, Molecular Devices, Sunnyvale, CA, USA). Electrodes were electrically connected to a patch-clamp amplifier (RK-400, Bio-logic Science Instruments, Claix, France).

For giant-patch experiments, the procedure has been described previously [24]. An ‘excision’ pipette, filled with the standard solution, was connected to a 10 ml syringe to apply suction for excision. Pipettes were pulled from borosilicate glass capillaries (glass type 8250; Garner Glass) on a vertical puller (P30; Sutter Instruments Co., Novato, CA, USA) and fire polished using a microforge (MF-83; Narishige, Japan) to reach 9 to 12 µm tip diameters for patch pipettes and around 15 µm for excision pipettes. Cells were continuously perfused with the standard solution. KCNE1-KCNQ1 currents were analyzed using a protocol consisting of depolarizing voltage steps of 1 s from a holding potential of −80 mV to +80 mV and then to −40 mV for 0.5 s, every 5 or 2 s. A microperfusion system allowed local application and rapid change of the different experimental solutions.

For permeabilized-patch experiments, micropipettes (tip resistance: 2–3 MOhms) were pulled from soda lime glass (Kimble; Vineland, New Jersey, USA). KCNE1-KCNQ1 currents were investigated with a protocol consisting of depolarizing voltage steps of 4 s from a holding potential of −80 mV to +80 mV and then to −40 mV for 1 s, every 7 s. To obtain the WT activation curve, the membrane potential was stepped, from a holding potential (−80 mV) to six voltage steps (−20 to 80 mV, with a 20 mV increment) and then stepped back to −40 mV, where tail currents are visible. To obtain the R539W activation curve, the membrane potential was stepped, from a holding potential (−80 mV) to six voltage steps (20 to 120 mV, with a 20 mV increment) and then stepped back to −40 mV, where tail currents are visible. Activation curves were fitted by a Boltzmann equation. KCNE1-KCNQ1 deactivation kinetics were obtained by a monoexponential fit.

For ruptured-patch experiments (Ci-VSP), the same pipettes as for permeabilized-patch experiments were used. KCNE1-KCNQ1 currents were investigated with a protocol consisting of depolarizing voltage steps of 2 s from a holding potential of −80 mV to +80 mV and then to −40 mV for 1 s, every 8 s.

Patch-clamp data are presented as mean ± SEM. Statistical significance of the observed effects was assessed by one-way ANOVA or Student's t-tests. Off-line analysis was performed using Acquis1, Clampfit and Microsoft Excel programs. Microsoft Solver was used to fit data by a least-square algorithm.

### Solutions and drugs

For giant-patch experiments, the cells were perfused with a standard solution containing (in mmol/L): 145 KCl, 10 HEPES, 1 EGTA (pH 7.3 with KOH). The following solution (in mmol/L): 145 K-gluconate, 10 HEPES, 1 EGTA, (pH 7.3 with KOH) was used to perfuse the cell during K^+^ current measurements and to fill the patch pipette tip (the Cl^−^-containing solution was in contact with the Ag^+^/AgCl filament).

For permeabilized-patch experiments, the pipette (intracellular) solution had the following composition (in mmol/L): 145 KCl, 10 HEPES, 1 EGTA and 0.85 amphotericin B (pH 7.2 with KOH). The Tyrode superperfusion solution contained (in mmol/L): 145 NaCl, 4 KCl, 1 CaCl_2_, 1 MgCl_2_, 5 HEPES, 5 glucose (pH 7.4 with NaOH). The locally applied extracellular control solution (334 mosmol/L) contained (in mmol/L): 145 NaCl, 4 KCl, 1 CaCl_2_, 1 MgCl_2_, 5 HEPES, 5 glucose, 20 mannitol (pH 7.4 with NaOH). Hypoosmotic challenge (234 mosmol/L) was induced by a decrease of NaCl from 145 mmol/L to 86 mmol/L and an increase of mannitol from 20 mmol/L to 39 mmol/L. In hyperosmotic solution (434 mosmol/L) NaCl was increased to 205 mmol/L and mannitol was decreased to 0.9 mmol/L. We looked at the effect of osmolarity on R539W and WT channels in the same set of experiments. The effects on the WT channel have been previously published and serve as a positive control [14]. For ruptured-patch experiments, the pipette (intracellular) solution had the following composition (in mmol/L): 145 KCl, 10 HEPES, 1 EGTA and 1 MgCl_2_ (pH 7.2 with KOH). Cells were perfused with Tyrode solution.

Available PIP_2_ was decreased by three ways: in the permeabilized-patch configuration, PIP_2_ was decreased by application of 10 µmol/L wortmannin [16], [25], [26]. In giant-patch experiments, available PIP_2_ was decreased by direct application of 1.1 mmol/L free Mg^2+^ on the intracellular side of giant patches [14]. In ruptured-patch configuration, channels were coexpressed with the voltage-dependent phosphatase from *Ciona intestinalis*, Ci-VSP, allowing PIP_2_ dephosphorylation [27], [28].

In channel PKA-dependent phosphorylation experiments, cells were exposed to a solution containing (in µmol/L) 400 cpt-cAMP, 10 forskolin and 0.2 okadaic acid. For cholesterol depletion experiments, two different approaches were used: one-hour pre-treatment of 2 mmol/L 2-hydroxypropyl-β-cyclodextrin in Tyrode solution at 37°C or twenty-four hour pre-treatment of 10 µmol/L triparanol [6] in 1 ml of the cell culture medium. Free magnesium activities in gluconate-containing solutions were calculated using software designed by G. L. Smith (University of Glasgow, Scotland), using stability constants [29].

### Molecular Modeling

#### Peptides

3D models of the WT and mutated sequence fragments, QQARKPYDV-R539-DVIEQYSQG and QQARKPYDV-W539-DVIEQYSQG, were calculated using Peplook [30]. Briefly, populations of 3D models are built from sequences using a boltzmanian stochastic procedure in which calculated populations are 3D models of lower energy. Models are all-atoms and their energies take into account all interactions between non-bound atoms with a cut-off of 20 Å. Energy terms are van der waals (Lennard-Jones), electrostatic (coulomb with an exponentially variable dielectric constant from 1 to 80 on a distance of 2 to 30 Å) and two hydrophobicity terms, one for intramolecular interactions and the second for solvent effect. The first term uses atomic transfer energy (Etr) and atom distances, the second one uses atom accessible atom surface area and atom Etr in the corresponding solvent, as previously described [30]. The best model is named the Prime and the next 98 models of low energy are also sorted to form the peptide population. The populations are clustered around lead models on the basis of backbone RMSd<1 Å.

#### Lipids

3D models of lipids were calculated by the same procedure as PepLook but using a dataset of rotation angles covering the 360° rotation possibilities by steps of 7.5°.

#### Impala

The Prime models of each peptide and lipid were individually tested across the water/membrane slab named IMPALA to define their best position [31]. IMPALA is a continuous restraint potential mimicking membrane hydrophobicity, charge density and fluidity properties with respect to water. The mass centre of each model was set at 101 different levels *i.e.* every angstrom from −50 to +50 from the membrane centre, across the slab. At each position, the restraint potential of 10,000 different orientations (rotations in the x/y plane of the membrane) was calculated and used to select the best position. In the figures, colored grids indicate different membrane interfaces: pink, the water/membrane interface; purple, the polar head/acyl chain interface of lipids; yellow, the membrane centre.

#### Molecule hydrophobicity and charge

To illustrate molecule hydrophobicity and charge, we used MHP (Molecular Hydrophobicity Potential) and MEP (Molecular Electrostatic Potential). MHP and MEP are three-dimensional plots of the hydrophobicity and electrostatic isopotential surface of a molecule, respectively [32]. In the MHP plots, surfaces joining all points of ±0.1 kcal are drawn; green for the hydrophilic surface, brown for the hydrophobic surface. In the MEP plots, surfaces joining all points of ±10 kcal are drawn; blue for the negatively charged surface, red for the positively charged surface.

## Results

### R539W KCNE1-KCNQ1 is poorly sensitive to PIP_2_ decrease

To obtain a quantification of channel PIP_2_ sensitivity, addition of soluble diC8-PIP_2_ is commonly used [8]. In a previous study, we used this approach to determine the PIP_2_ sensitivity of three LQT1-associated KCNQ1 channels containing the R243H, R539W or R555C mutation [10]. However, this approach may be limited because, for some channels, the maximum effect of short-chain PIP_2_ is less than that of PIP_2_, suggesting a lower affinity [33] and/or a contribution of the acyl chain to the channel-PIP_2_ interaction. As an extreme example, the Kv11.1 channel is sensitive to PIP_2_ but not to diC8-PIP_2_ [21]. These new results prompted us to use alternative methods to confirm the decreased PIP_2_ sensitivity of R243H, R539W and R555C mutant channels. We used three different approaches: Extracellular wortmannin effects on whole-cell currents, intracellular magnesium effects on excised-patch currents and Ci-VSP-coexpression effects on whole-cell currents [10], [18], [27], [28], [34].

**Figure 1:**
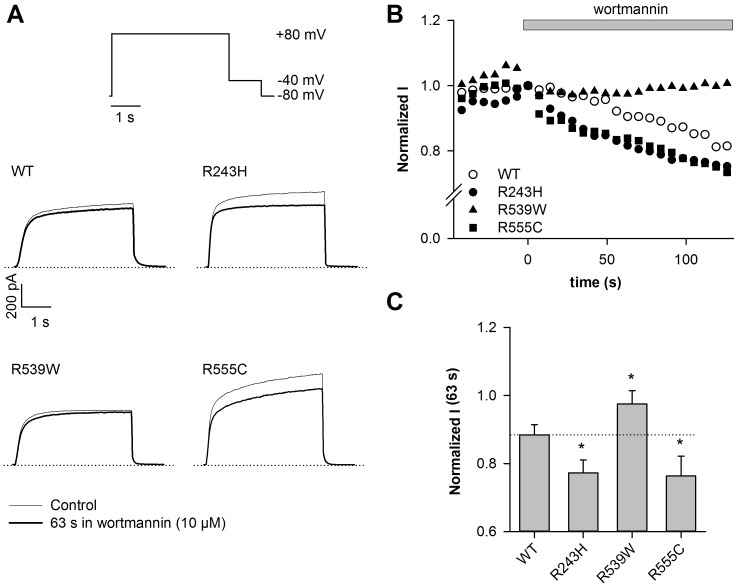
R539W is insensitive to wortmannin. A, representative permeabilized-patch current recordings of WT or mutant channels measured before or after a 63-s wortmannin application (10 µmol/L), and during the voltage-clamp protocol shown. Start-to-start interval = 7 s. B, relative current amplitude of WT or mutant channels measured at the end of the depolarizing step (+80 mV), plotted against time. Current values are normalized to the current level measured before wortmannin application (time 0). These experiments were performed at 35°C in the permeabilized-patch configuration. In this configuration, it has been shown that there is no spontaneous current rundown [24]. C, mean relative current amplitude of WT or mutant channels measured after a 63-s wortmannin application (n = 5–7). *p<0.05, *versus* WT.

In the permeabilized-patch configuration, we monitored the activity of WT and mutant KCNE1-KCNQ1 channel currents during application of 10 µmol/L wortmannin that blocks the PI4-kinase required for PIP_2_ synthesis. Current density measured at the end of the depolarizing step (+80 mV) was monitored every 7 s and normalized to the current value measured before wortmannin application (time 0). Wortmannin application led to a gradual decrease of WT KCNE1-KCNQ1 channel activity, called rundown (Figure 1A and 1B). This rundown is also significant for the R243H and R555C, but not for the R539W channel (Table S1 in File SI). In order to compare the rundown kinetics in the different conditions, the relative current amplitude after 63 s in 10 µmol/L wortmannin was calculated. Rundown was accelerated for R243H and R555C mutants as compared to WT (Figures 1B and 1C), confirming a decreased PIP_2_ sensitivity. Relative current amplitude after 63 s in 10 µmol/L wortmannin was 0.88±0.03 (n = 7) for WT channel, and 0.77±0.04 (n = 5; *P*<0.05) and 0.76±0.06 (n = 5; *P*<0.05) for R243H and R555C mutants, respectively. Most importantly, despite the lower affinity of short-chain PIP_2_ for R539W, the decrease of PIP_2_ levels caused by wortmannin application had no effect on this mutant channel activity (Figures 1B and 1C).

**Figure 2:**
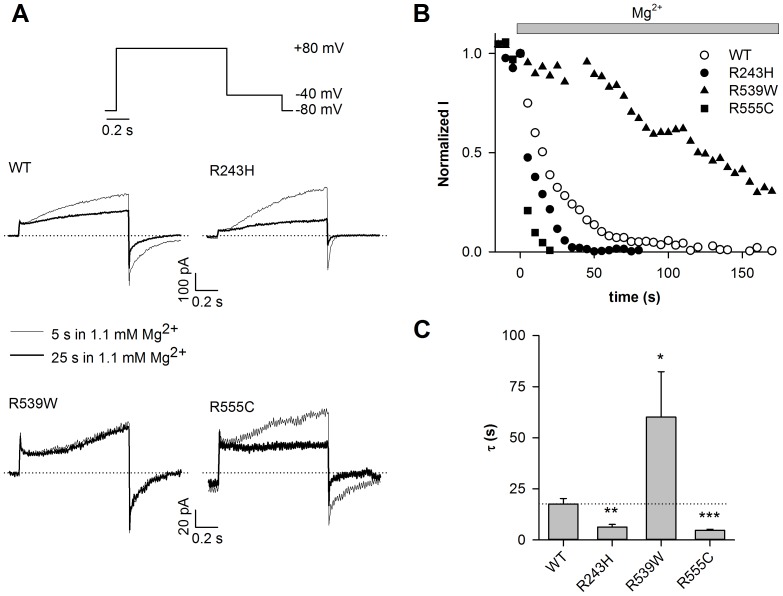
R539W is poorly sensitive to intracellular magnesium. A, representative giant-patch current recordings of WT or mutant channels measured after 5 and 25-s Mg^2+^ application (1.1 mmol/L free Mg^2+^), and during the voltage-clamp protocol shown. Start-to-start interval = 5 s. B, relative tail-current amplitude (measured at −40 mV) of WT or mutant channels after a depolarization to +80 mV, plotted against time. Current values are normalized to the current level measured before magnesium application (time 0). C, rundown time constant (τ) of WT and mutant channel currents (n = 8–12). *p<0.05, **p<0.01, ***p<0.001 versus WT.

We tried to decrease PIP_2_ intracellular levels to a higher extent, in an attempt to decrease R539W channel activity. To do that, we used intracellular Mg^2+^, which is known to mask PIP_2_ negative charges [35]. We recorded WT and mutant channels activities during 1.1-mmol/L free Mg^2+^ application using the excised-patch configuration (Figure 2, Table S2 in File SI). As previously shown, Mg^2+^ application led to a gradual decrease of WT KCNE1-KCNQ1 channel activity (Figure 2A and 2B; τ = 18±3 s, n = 12). R243H and R555C currents decreased faster than that of WT, as during wortmannin application (Figure 2B and 2C; R243H, τ = 6.3±1.3 s, *P*<0.01 and R555C, τ = 4.7±0.5 s; n = 10–12, *P*<0.001 as compared to WT). Conversely, the R539W channel current ran down more slowly than the WT (τ = 60±22 s; n = 8; *P*<0.05).

**Figure 3:**
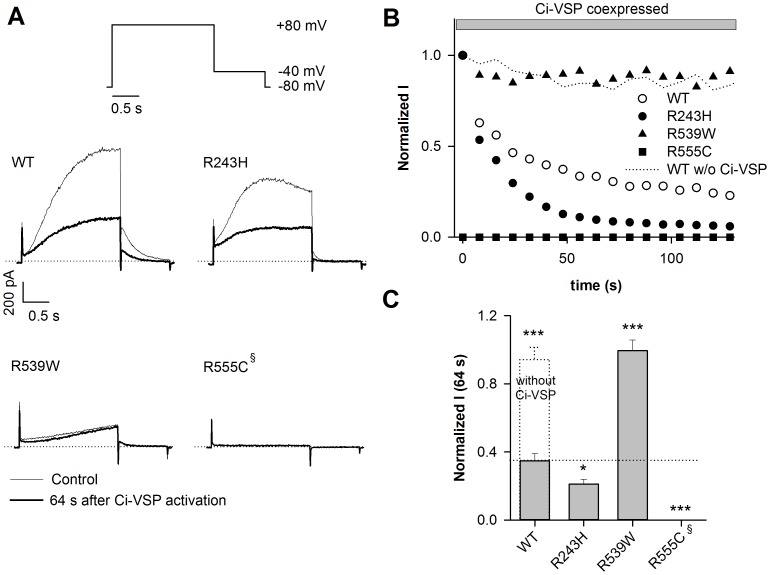
R539W is insensitive to Ci-VSP. A, representative ruptured-patch current recordings of WT or mutant channels coexpressed with the voltage-dependent membrane phosphatase, Ci-VSP, at the first (t = 0 s) and 9^th^ (t = 64 s) step of depolarization. These currents were measured during the voltage-clamp protocol shown. Start-to-start interval = 8 s. The +80-mV depolarization also allows Ci-VSP activation. B, relative tail-current amplitude (measured at −40 mV) of WT or mutant channels after a depolarization to +80 mV, plotted against time. Current values are normalized to the current amplitude measured before Ci-VSP activation (time 0). C, mean relative current amplitude of WT or mutant channels measured after a 64-s Ci-VSP activation (n = 6–11). *p<0.05, ***p<0.001 versus WT. ^§^R555C already ran down before Ci-VSP activation, due to basal Ci-VSP activity, it is thus assimilated to 0 (n = 18). WT condition without Ci-VSP is shown in (B) and (C).

To confirm these results, we depleted PIP_2_ by coexpressing the channels with the voltage-dependent phosphatase Ci-VSP, which is known to dephosphorylate PIP_2_ under membrane depolarization [27]. Ci-VSP activation led to a gradual decrease of WT KCNE1-KCNQ1 channel activity (Figure 3A and 3B). As a negative control, the WT KCNE1-KCNQ1 channel was expressed without Ci-VSP and no current rundown was observed (Figure 3, Table S3 in File SI). Tail current density measured at −40 mV was monitored and normalized to the current value measured at time 0. The relative current amplitude after 64 s of Ci-VSP activity was calculated. Rundown was accelerated for R243H mutant as compared to WT (Figure 3B), confirming a decreased PIP_2_ sensitivity. The relative current amplitude after 64 s of Ci-VSP activity was 0.35±0.04 (n = 11) and 0.21±0.02 (n = 6; *P*<0.05) for WT and R243H, respectively. When coexpressed with Ci-VSP, the R555C mutant channel had a very low current density (at time 0, I_tail_ = 0.16±0.08 pA/pF, p<0.001, n = 18) as compared to the mutant channel activity without Ci-VSP coexpression (I_tail_ = 4.27±1.64 pA/pF, n = 4). Such a Ci-VSP coexpression had no effect on the WT current density (I_tail_ = 13.4±7.64 pA/pF, n = 7 and I_tail_ = 25.4±6.15 pA/pF, n = 11 without and with Ci-VSP respectively, N.S.). Consistent with the lower affinity of the R555C mutant to diC8-PIP_2_ [10], these results suggest that Ci-VSP has enough basal activity (i.e. without any depolarization to +80 mV) to decrease membrane-PIP_2_ levels and that such a basal PIP_2_ decrease is able to affect the R555C channel current density without any effect on the WT channel. In contrast, the decrease of PIP_2_ levels caused by Ci-VSP activation had no effect on R539W channel activity (normalized I at 64 s = 0.99±0.06, n = 9, p<0.001 *versus* WT; Figure 3C).

These three different approaches show that, contrary to R243H and R555C mutants, R539W is much less sensitive to variation in PIP_2_ levels than WT. *A priori*, the results are inconsistent with the similar sensitivity of R539W and R555C to diC8-PIP_2_ observed previously [10]. The impaired Mg^2+^, wortmannin and Ci-VSP effects on R539W channel rundown questioned the validity of these approaches in the evaluation of PIP_2_ sensitivity. We will address, later in this article, why the R539W channel is less sensitive to PIP_2_ variation than WT.

### R539W is insensitive to osmolarity

**Figure 4:**
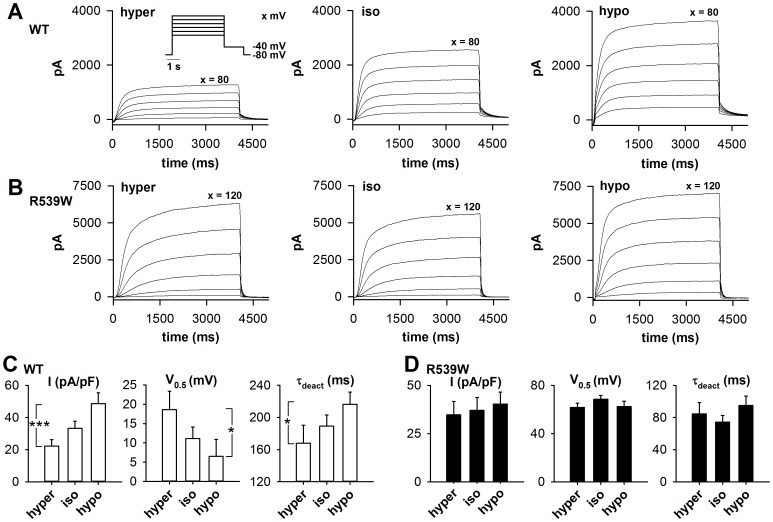
R539W is insensitive to osmolarity. A, B, superimposed representative permeabilized-patch recordings of WT (A) and R539W (B) KCNE1-KCNQ1 concatemer currents, respectively, measured in hyper-, iso- and hypoosmotic conditions using the voltage protocol shown in the insert. C, D, averaged tail-current density (in pA/pF), V_0.5_ (in mV) and τ_deact_ (in ms) measured at −40 mV after a depolarization step to +80 mV for WT (C) and +120 mV for R539W (D) channel, in hyperosmotic (hyper), control (iso), and hypoosmotic solutions (hypo) (n = 10); same voltage protocol as in A. *p<0.05, ***p<0.001 (one-way ANOVA for repeated measures). Protocol and experimental conditions are similar to those used in our previous study analyzing the osmoregulation of KCNE1-KCNQ1 [14].

We then studied the R539W channel response to a physiological range of PIP_2_ variation, by varying extracellular osmolarity. We previously showed that switching from hyperosmolar to hypoosmolar extracellular solution leads to an increase in available membrane PIP_2_, provoking an increase in the WT KCNE1–KCNQ1 current density, a shift of the activation curve towards negative potentials and slower deactivation kinetics (Figure 4A, 4C and [14]). During the same set of experiments, we measured R539W channel current in isoosmolar (334 mosmol.L^−1^), hypoosmolar (234 mosmol.L^−1^, 70% of control osmolarity) and hyperosmolar extracellular solutions (434 mosmol.L^−1^, 130% of control osmolarity; cf. Methods). Tail current density (measured after a +120-mV depolarization), half-activation potential and time constant of deactivation at −40 mV (τ_deact_) were changed neither by switching from isoosmolar to hypoosmolar condition, nor by switching from isoosmolar to hyperosmolar condition (Figure 4B and 4D). This lack of R539W channel osmoregulation is supporting the idea that the mutant is much less sensitive to variations in PIP_2_ levels than the WT channel.

### R539W is as sensitive to PKA as WT KCNE1-KCNQ1

**Figure 5:**
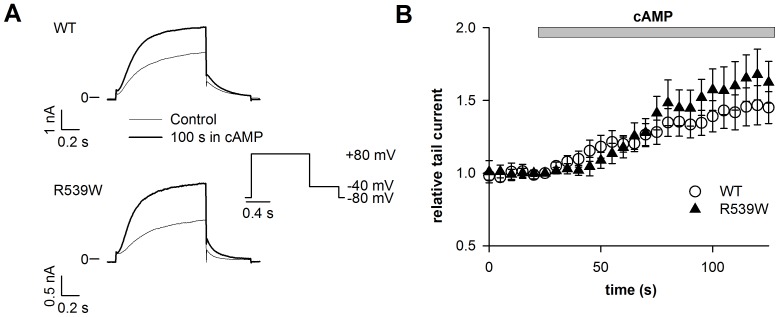
R539W is sensitive to PKA. A, representative permeabilized-patch current recordings of WT or R539W channels (co-transfected with yotiao) measured during the voltage-clamp protocol shown. B, mean time course of channel activation by 400 µM cpt-cAMP, 10 µM forskolin and 0.2 µM okadaic acid (cAMP) for WT or R539W KCNE1-KCNQ1 concatemer channel tail currents measured at −40 mV after a 1 s depolarization to +80 mV and normalized to the current value before cAMP application (n = 12–13).

For some Kir channels, it has been established that phosphorylation by PKA increases their interactions with PIP_2_ [9], [19]. Several studies have shown that KCNQ1 activity is regulated by PKA-dependent phosphorylation [36]–[38] and two studies suggested that the PKA-dependent phosphorylation of KCNQ1 increases its interaction with PIP_2_ [9], [11]. If so, PKA should have less impact on R539W KCNE1-KCNQ1 current since R539W is less sensitive to PIP_2_ variation. To test this idea, cells expressing KCNQ1, KCNE1 and the PKA-anchoring protein yotiao, were exposed to a solution containing (in µmol/L) 400 cpt-cAMP, 10 forskolin and 0.2 okadaic acid. Tail current density after a 1-s depolarization to +80 mV was measured in the permeabilized-patch configuration. cAMP had a similar effect on normalized tail currents of the WT and of the R539W channel (Figure 5; WT, 1.45±0.11 and R539W, 1.63±0.14; n = 12–13), indicating that the R539W mutant channel is as sensitive to PKA-dependent phosphorylation as the WT channel. Therefore, PKA regulation of KCNE1-KCNQ1 channel does not seem to act through a modulation of channel-PIP_2_ interaction, which is consistent with the recent publication of Li et al, where phospho-mimetic mutations do not affect PIP_2_ dependent rundown [12].

### Hypothesis on the molecular basis of the low sensitivity to PIP_2_ variation of R539W

R539W and R555C mutants have fully-activated current amplitude similar to the WT channel [10], supporting the conclusion that they are similarly expressed, correctly folded, and fully processed to the cell membrane. R539W and R555C mutants have similar sensitivity to diC8-PIP_2_ on one hand [10], but different responses to a decrease in membrane PIP_2_ on the other hand (Figures 1, 2 and 3). Since a decrease in membrane PIP_2_ closes the WT channel [13], our data indicate that the R539W channel is steadily open and poorly sensitive to membrane PIP_2_ variation. Thus, R539W open state is not or less dependent on a direct or indirect interaction with PIP_2_, as compared to WT.

To better understand the mechanism by which R539W is desensitized to variations in PIP_2_ levels, we used molecular modeling to analyze the structure of the WT and mutant QQARKPYDV-R/W539-DVIEQYSQG fragments of the KCNQ1 C-terminus and their potential interaction with the membrane bilayer. In the absence of a full 3D model of the channel, only partial models were used to reach a working hypothesis.

**Figure 6:**
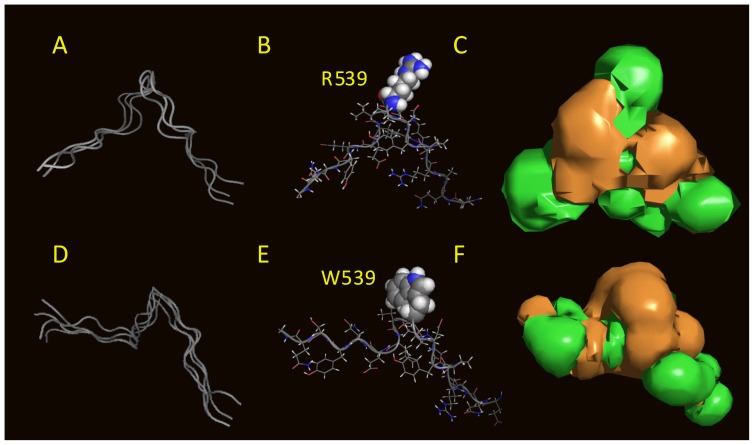
PepLook models of the 19-aa sequence surrounding R539 and W539. The sequences QQARKPYDVR539DVIEQYSQG and QQARKPYDVW539DVIEQYSQG were used to calculate amphipathic 3D structure models in water. A, D, the 99 PepLook models of low energy were clustered into three WT (A) and four R539W (D) lead models on the basis of backbone RMSd<1 Å. Ribbon structures of these lead models are fitted in (A and D) demonstrating their large similarity. B, E best models (Prime), with R539 in CPK for the WT fragment (B) and W539 in CPK for the mutant fragment (E). Both residues are protruding in a pin loop-like structure. C, F, hydrophobicity profiles of the WT and R539W Primes are visualized by the hydrophobic (brown) and hydrophilic (green) isopotential surface (+0.1 kcal/mol) around the molecule. The MHP profiles demonstrate, first, the rather important hydrophilicity of the models, and second, that changing R to W, transforms a very polar protuberance to an apolar one.

**Figure 7:**
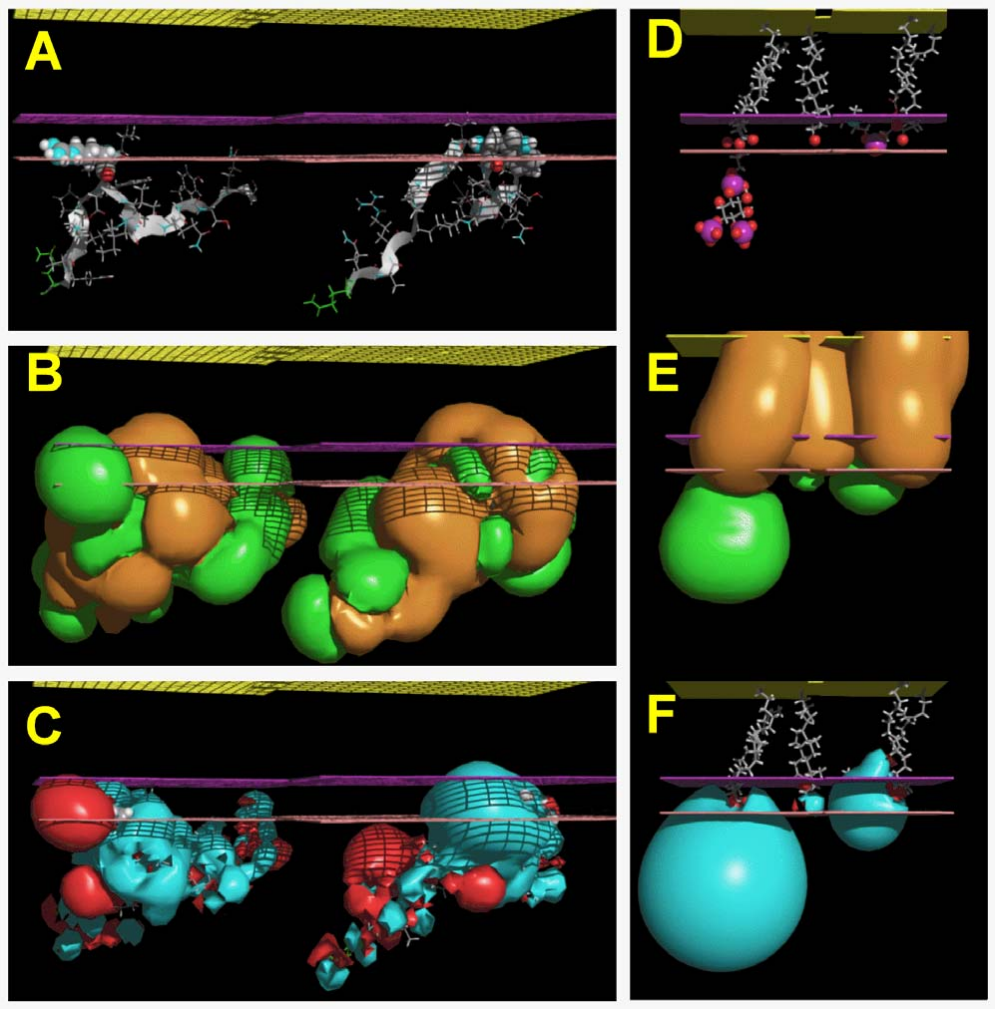
Relative position of a series of molecules in the membrane. All molecules are shown at their optimal position in the IMPALA slab after they were systematically tested at every Å across a water/membrane continuous layer. In all plots, the yellow grid represents the membrane center; the purple grid, the averaged lipid head/acyl chain interface, and the pink grid the averaged lipid/water interface. A, PepLook model of the WT (left) and R539W (right) peptides. In both cases, residue 539 is imbedded in the membrane. B, MHP (Molecular Hydrophobicity Potential) profiles of fragments. R539 is responsible for a large hydrophilic (green) protuberance, whereas W539 is a hydrophobic (brown) protuberance in the membrane. C, MEP (molecular electrostatic profile) of the same molecules showing the e-attractivity of the R539 protuberance. D, E and F, same as in A, B and C except that molecules are, from left to right, an extended fluid form of PIP_2_, cholesterol, and a fluid form of DOPC. They highlight the relative position of PIP_2_ and peptides and the fact that the more hydrophobic cholesterol is embedded in the membranes.

Three dimensional structures of the fragments were calculated with PepLook [30]. For both fragments the 99 lower energy models were sorted and compared. For the WT sequence, the 99 models had a similar conformation *i.e.* a β-extended polar hairpin. R539 was located in the U-turn (Figure 6B). Models were clustered into three leads on the basis of RMSd less than 1 Å (Figure 6A). Leads were stabilized by aromatic (Y536/Y545) side chain interactions or by NH…π and NH…O H-bonds but mainly by side chain (R533-E543 and/or K534-D540) salt bridges. Of note, mutations of R533 and E543 are associated with LQT1 [39], consistent with their role in the hairpin structure stabilization. The lead models were polar structures with a mean hydrophobic to hydrophilic accessible surface ratio of 0.73±0.20 (Figure 6C). Because of its hydrophilicity, the fragment should map to the channel protein surface rather than in its core, being accessible to external partnership. In addition, due to its high content in positively-charged residues (R533, K534 and R539) and especially to the fact that R539 was accessible in all models, the fragment might be able to interact with negative charges of membrane phospholipids. As a basis for this hypothesis, we found that the best position of the WT fragment models was to be adsorbed on the membrane surface, with R539 diving in the interface (Figure 7A). In that position, what could be the R539 membrane partner? In Figure 7D, we show the optimal positions of PIP_2_, cholesterol and DOPC in the same membrane slab. The depth of insertion of the WT fragment models argues for a possible interaction of R539 with the C = O moiety of PIP_2_ (Figure 7D–7F).

For the R539W mutant, changing R to W changed the 3D structure but not the extended conformation (Figure 6A compared with 6D). Four lead models were sorted (Figure 6D) and W539, like R539, was a structural protuberance. The hydrophobicity profiles of the WT and R539W models demonstrate that changing R to W decreased the hydrophilicity (Figure 6F to be compared with Figures 6C, 7B and 7C). The R539W mutant had a mean hydrophobic to hydrophilic accessible surface ratio of 0.81+0.10 supporting the conclusion that the fragment was still polar and that the R539 polar protuberance of the native fragment was now a W539 hydrophobic protuberance. The best position of the R539W mutant channel fragment in the membrane/water slab was the membrane interface similar to the WT fragment (Figures 7A–7C). Even though fragment positions are similar, their partners in the membrane should be different. The WT fragment could have a polar R-phospholipid interaction whereas the mutant fragment would more likely favor an interaction of the apolar W539 with a more hydrophobic partner.

Among natural membrane lipids, cholesterol has been implicated in direct interactions with some channels and in the regulation of their activities [40], [41]. Various studies have suggested that protein-cholesterol interactions implicate tryptophan [42], [43]. Interestingly, in another ion channel, a mutation of a cysteine to a tryptophan in a lipid-exposed position enhances the effect of cholesterol [42]. Tryptophan can interact with cholesterol in a rigid cycle-to-cycle hydrophobic fit stabilized by OH (cholesterol) - π (tryptophan cycle) or by NH (tryptophan cycle) - O (cholesterol) interactions if the two partners are at the same depth of insertion in the membrane. Our calculations indicate that the cholesterol polar head and tryptophan are at the same level in the membrane and thus justify the hypothesis that changing R539 to W could switch a polar R-PIP_2_ interaction to a W-cholesterol interaction.

### R539W interacts with membrane cholesterol

To test this hypothesis, we probed whether the decreased sensitivity of the R539W mutant to PIP_2_ was associated with a gain in cholesterol sensitivity. We tried three different approaches: first, we measured the Mg^2+^-induced current rundown of the R539F KCNE1-KCNQ1 channel, phenylalanine being less hydrophobic and bulky than tryptophan and thus likely to be a less efficient membrane anchor than tryptophan. Second, we compared the effect of depleting the membrane cholesterol by 2-hydroxypropyl-β-cyclodextrin (cyclodextrin) on WT and R539W channel current [44]. Since cyclodextrin is not very specific, we used another way of depleting membrane cholesterol. We selected triparanol because it has been shown to decrease cholesterol in a similar model [6]. In all these approaches we measured the relative tail-current amplitude of WT and mutant channels after a depolarization to +80 mV, during 1.1-mmol/L free Mg^2+^ application in the giant-patch configuration.

**Figure 8:**
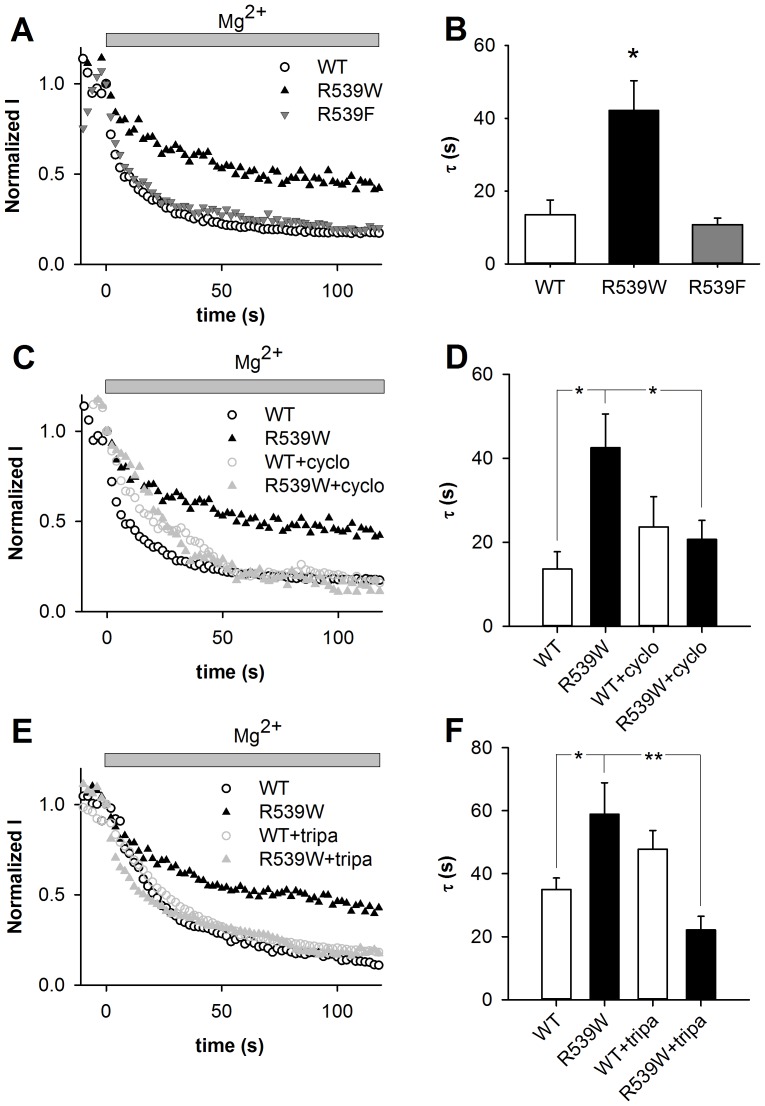
Effect of intracellular magnesium on WT, R539W and R539F mutant channels, and after membrane cholesterol depletion. A, C, E, relative tail-current amplitude (at −40 mV) of WT or mutant channels measured after a depolarization to +80 mV plotted against time during a 1.1 mmol/L free Mg^2+^ application on a giant-patch. Start-to-start interval = 2 s. Current values are normalized to the current level measured before magnesium application (time 0). Cholesterol depletion was induced by 1 hour of 2 mmol/L cyclodextrin (cyclo) or 24 hours of 10 µmol/L triparanol (tripa) pre-treatment. B, D, F, mean rundown time constant (τ) of WT and mutant channels (B), and with and without 2 mmol/L cyclodextrin (D, n = 9–13) or 10 µmol/L triparanol (F, n = 9–15). *p<0.05, **p<0.01 *versus* WT. DMSO in which triparanol was diluted (E and F) has an effect on rundown kinetics (τ = 15.4±1.6, n = 7 *versus* τ = 35.0±3.65, n = 15, without and with DMSO pre-treatment respectively, p<0.01).

As expected, the Mg^2+^-induced current rundown of the R539F mutant channel was faster than the rundown of the R539W mutant channel activity and as fast as the rundown of WT current (Figures 8A and 8B). This behavior is consistent with a lower anchoring of the F539 on the membrane as compared to that of W539. The fact that R539F mutant channel is not running down faster than WT suggests that phenylalanine still interacts with cholesterol although less than the tryptophan does.

Second, we compared the Mg^2+^-induced rundown of the R539W mutant current without and with 2 mmol/L of cyclodextrin. As shown in Figures 8C and 8D, cyclodextrin pre-treatment accelerated the R539W rundown. Thus, when membrane cholesterol is decreased, the R539W channel is more sensitive to PIP_2_ variation. In WT channels, only activation kinetics were affected by cholesterol depletion, as shown in Figure S1 in File SI and consistent with a previous work [6], but rundown was not affected (Figure 8C and 8D). As opposed to WT channels, R539W activation kinetics were not modified by cyclodextrin (Figure S1 in File SI).

Finally, we compared the Mg^2+^-induced rundown of the R539W mutant current without and with 10 µmol/L of triparanol. Like cyclodextrin, triparanol pre-treatment accelerated the R539W rundown without any significant change in the WT rundown (Figure 8E and 8F). These results confirm that the R539W channel is more sensitive to PIP_2_ variation when membrane cholesterol is decreased. In addition, as for cyclodextrin, in WT channels, only activation kinetics were affected by the triparanol pre-treatment. In R539W mutant channels, activation kinetics were unchanged by the triparanol pre-treatment (Figure S2 in File SI), consistent with the results using cyclodextrin to deplete membrane cholesterol. These results reinforce the idea that interaction of cholesterol with WT and R539W channels is quite different.

Altogether, data from experiments manipulating the residue at position 539, and cholesterol levels in the membrane, are in agreement with the hypothesis that the tryptophan in position 539 interacts (or induces an interaction) with membrane cholesterol. This channel-cholesterol interaction might overrule the channel-PIP_2_ interaction to stabilize the channel open-state.

## Discussion

In previous studies, we have shown that KCNE1-KCNQ1 channel open-state is stabilized by PIP_2_ and impairment of this stabilization by arginine neutralization at position 243, 539 or 555 in KCNQ1 is correlated with the long QT syndrome [10], [13]. For the three mutants, higher diC8-PIP_2_ concentrations than for WT were needed to stabilize the open state after the channel activity had run down, suggesting a decrease in interaction with PIP_2_ [10]. The R539W and R555C mutations are localized in the cytosolic C-terminus (CTD) [45]. The supposed interaction of arginines 539 and 555 with PIP_2_ suggests that they are situated on the membrane-cytosol interface, which may be surprising since they are located in the middle of the distal half of the KCNQ1 cytosolic CTD [45]. In a recent crystallographic study, Hansen et al. described that PIP_2_ mediates docking of the whole CTD to the transmembrane module and subsequent opening of the inner helix gate of the Kir2.2 channel [46]. Thereby, the KCNQ1 distal CTD might come close in order to interact with the membrane, *via* interactions such as R539-PIP_2_ and R555-PIP_2_, allowing the CTD to be in the vicinity of the pore domain for modulating its opening. However, further crystallographic studies should address this hypothesis.

In the present study, the most striking result was that decreasing endogenous PIP_2_ has a very small effect on the open-state stability of the R539W mutant. R539W is paradoxically less sensitive to a decrease in PIP_2_ than WT. This is in contrast with many channel mutations that reduce their affinity for PIP_2_ or diC8-PIP_2_ [47], [48], including the two other KCNQ1 mutations R243H and R555C. Here we suggest that the paradoxical behavior of R539W is due to the stabilizing effect of tryptophan-cholesterol interaction that replaces the arginine-PIP_2_ interaction. Indeed, severe cholesterol depletion of the membrane restored the PIP_2_ sensitivity of the channel. After cyclodextrin or triparanol pre-treatment, R539W rundown kinetics during Mg^2+^ application were restored to values similar to WT, suggesting that membrane cholesterol depletion abolished the R539W open pore stabilization, now only maintaining its open stabilization through other PIP_2_-interacting residues (such as R243 and R555). It is important to note that - when expressed in Xenopus oocytes - R539W behaved exactly as R555C [12]. In that model, both excision-induced rundown and PIP_2_ sensitivity were the same for R555C and R539W. This apparent inconsistency between models may be due to different cholesterol levels around the channel in X. oocytes *versus* COS-7 cells, with higher levels in the latter, slowing down R539W rundown.

For Kir4.1, Hibino and Kurachi showed that cyclodextrin abolishes the function of Kir4.1 channels in HEK293 cells [49]. One of the proposed mechanistic hypotheses suggests that cholesterol influences the conformation of Kir4.1 and activates it by specific protein-cholesterol interactions [49]. This hypothesis is supported by some studies performed on other channels. Singh et al. demonstrated a direct effect of cholesterol on the activity of KirBac1.1 [40]. They showed that changes in membrane fluidity could not account for the effects of the sterols on KirBac1.1 activity, and their data strongly pointed to a direct cholesterol-channel interaction. The present study is also supporting a direct and specific cholesterol-channel interaction.

We have previously reported that KCNE1-KCNQ1 activity is regulated by intracellular ATP in addition to PIP_2_ [13]. More precisely, Li et al. recently showed that ATP binds to the cytosolic domain and promotes pore opening [50]. Since the patch-clamp experiments were performed without ATP in the intracellular solution, the gradual decrease of channel activity is probably the sum of the PIP_2_- and the ATP-dependent channel rundown. This is especially true in giant-patch experiments, during which ATP is diffusing out of the patch very quickly. Even though the regulation of KCNQ1 by PIP_2_ and by ATP concerns two distinct mechanisms [13], and distinct binding sites [51], there may be some interplay between both types of regulation. For instance, it has been shown that variations in intracellular ATP provoke variations in membrane PIP_2_ levels [25], [52]. In that context, using R539W - which is much less PIP_2_-sensitive - may reveal to what extent PIP_2_-dependent rundown influences the ATP-dependent rundown.

KCNQ1 and KCNE1 localize in lipid rafts [53]–[55] as well as the G-protein-dependent machinery involved in β-adrenergic signaling [56]. From the literature, it appears that cholesterol may be involved in KCNQ1 expression levels and channel-complex trafficking [5], [57] and gating [6]. Here we show that cholesterol does not stabilize WT KCNE1-KCNQ1 channel opening *per se*. Substitution of R539 by W introduces a new sensitivity to acute cholesterol changes, possibly by favoring a direct binding of this residue to cholesterol, which is abundant in lipid rafts. In Kir2.1 channels, Epshtein et al. identified a specific region that plays a critical role in the sensitivity of these channels to cholesterol [58]. This region is in the CTD, consistent with the location of R539 in KCNQ1. Nonetheless, PIP_2_ still regulates partially the R539W mutant, since in the presence of cholesterol, severe depletion of PIP_2_ eventually induced open-state destabilization. This indicates that other PIP_2_ interacting residues (such as R243 and R555) are still needed for channel opening. This severe depletion of PIP_2_ allowed unmasking of the reduced diC8-PIP_2_ sensitivity of the R539W mutant, since higher doses of diC8-PIP_2_ are necessary to activate the channel [10].

We inferred the PIP_2_ sensitivity of WT and mutated channels from the measurement of the rundown kinetics of the corresponding current during a decrease in available PIP_2_.The decrease in PIP_2_ was triggered using three common approaches: extracellular wortmannin application on permeabilized-patch configuration, intracellular Mg^2+^ application on excised-patch configuration, and Ci-VSP coexpression on ruptured-patch configuration [10], [18], [27], [28], [34]. Although the R539W mutant showed a decreased affinity to diC8-PIP_2_ compared to WT [10], all these three approaches show that this mutant is less sensitive to a PIP_2_ decrease, which would classically suggest a higher affinity to PIP_2_. This peculiar behavior suggests that caution should be taken when using the current rundown in the evaluation of a channel PIP_2_ affinity, because, in some cases (e.g. R539W), the mutation that disrupts the interaction with PIP_2_ may stabilize the channel in the open state through another mechanism.

In conclusion, the present study shows that a channel mutation can induce or favor an interaction between the channel and a membrane component. We speculate that this PIP_2_-independent tryptophan-cholesterol interaction is responsible for the difference in phenotypes observed for patients with long QT syndrome caused by R539W and R555C mutations of KCNQ1. Until now, both mutations were characterized by similar functional effects in cellular models, but the R555C mutation is associated with a ‘forme fruste’ of type 1 long QT syndrome [59] whereas R539W is associated with cardiac sudden death [60]. This difference could be caused by the drastic decrease of sensitivity of the R539W mutant to PIP_2_ variation (cf wortmannin, Mg^2+^, osmolarity and Ci-VSP effects), and thus the poor sensitivity of the channel to signaling dependent on PLC-coupled receptors [61].

## Supporting Information

File S1 **Supporting Information Figures and Tables.** **Figure S1, Effect of cyclodextrin on WT and R539W activation kinetics.** A, Representative recording of a COS-7 cell transfected with WT or R539W KCNE1-KCNQ1 concatemer channel, without and with 1-hour pre-treatment by 2 mmol/L cyclodextrin. B and C, Mean +/− sem of WT and R539W activation tau from monoexponential fit of activation without and with cyclodextrin pre-treatment. * p<0.05. **Figure S2, Effect of triparanol on WT and R539W activation kinetics.** A, Representative recording of a COS-7 cell transfected with WT or R539W KCNE1-KCNQ1 concatemer channel, without and with 24-hour pre-treatment by 10 µmol/L triparanol. B and C, Mean +/− sem of WT and R539W activation tau from monoexponential fit of activation without and with triparanol pre-treatment. * p<0.05. **Table S1, Wortmannin-induced rundown. Table S2, Magnesium-induced rundown. Table S3, Ci-VSP-induced rundown.**
(PDF)
