# Supplementary material for: A Long QT Mutation Substitutes Cholesterol for Phosphatidylinositol-4,5-Bisphosphate in KCNQ1 Channel Regulation
Source: PLoS One. 2014 Mar 28;9(3):e93255. doi: 10.1371/journal.pone.0093255 (PMC3969324; doi:10.1371/journal.pone.0093255)
Supplement: File S1 — Supporting Information Figures and Tables. Figure S1, Effect of cyclodextrin on WT and R539W activation kinetics. A, Representative recording of a COS-7 cell transfected with WT or R539W KCNE1-KCNQ1 concatemer channel, without and with 1-hour pre-treatment by 2 mmol/L cyclodextrin. B and C, Mean +/− sem of WT and R539W activation tau from monoexponential fit of activation without and with cyclodextrin pre-treatment. * p<0.05. Figure S2, Effect of triparanol on WT and R539W activation kinetics. A, Representative recording of a COS-7 cell transfected with WT or R539W KCNE1-KCNQ1 concatemer channel, without and with 24-hour pre-treatment by 10 µmol/L triparanol. B and C, Mean +/− sem of WT and R539W activation tau from monoexponential fit of activation without and with triparanol pre-treatment. * p<0.05. Table S1, Wortmannin-induced rundown. Table S2, Magnesium-induced rundown. Table S3, Ci-VSP-induced rundown. (PDF) [file pone.0093255.s001.pdf]

Supplemental figures

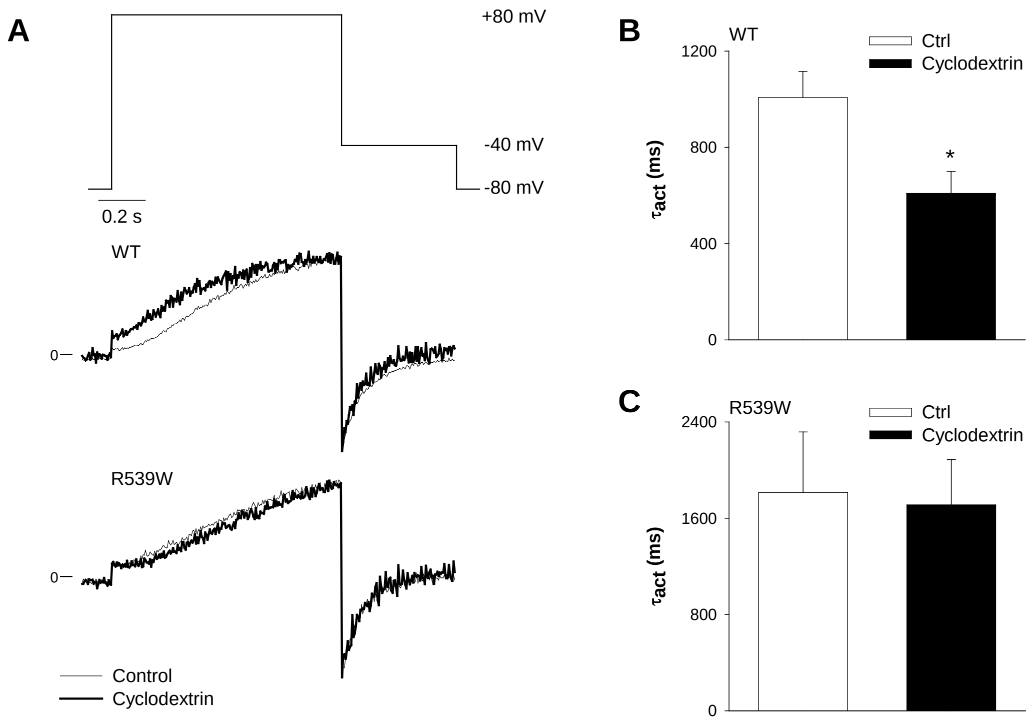

Figure S1

Effect of cyclodextrin on WT and R539W activation kinetics. A, Representative recording of a COS-7 cell transfected with WT or R539W KCNE1-KCNQ1 concatemer channel, without and with 1-hour pre-treatment by 2 mmol/L cyclodextrin. B and C, Mean +/- sem of WT and R539W activation tau from monoexponential fit of activation without and with cyclodextrin pre-treatment. * $p<0.05$.

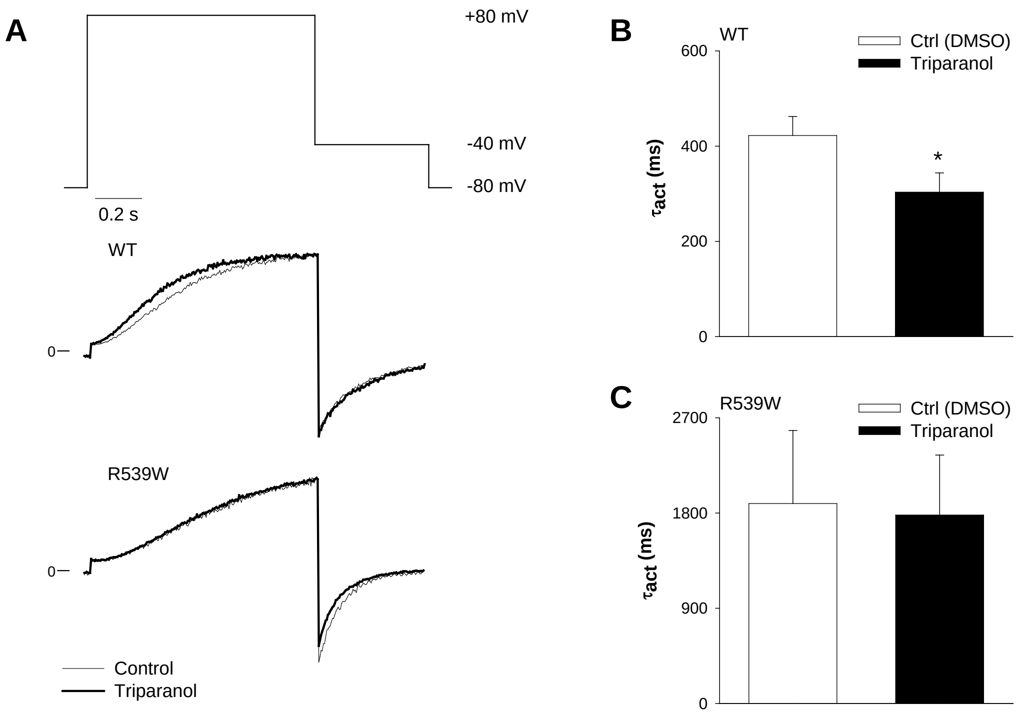

Figure S2

Effect of triparanol on WT and R539W activation kinetics. A, Representative recording of a COS-7 cell transfected with WT or R539W KCNE1-KCNQ1 concatemer channel, without and with 24-hour pre-treatment by 10 µmol/L triparanol. B and C, Mean +/- sem of WT and R539W activation tau from monoexponential fit of activation without and with triparanol pre-treatment. * $p<0.05$.

Supplemental tables

	WT		R243H		R539W		R555C	
Time (s)	0	63	0	63	0	63	0	63
Mean of current density (pA/pF)	112	98.4	40.7	31.8	85.5	83.3	63.0	52.7
SEM	21.0	17.3	13.1	10.8	13.2	13.0	27.6	24.0
n	7	7	5	5	7	7	5	5
p value (one-tail paired t-test)	0.03		0.02		0.24		0.03	
Significant difference	YES		YES		NO		YES	

Table S1: Wortmannin-induced rundown.

	WT		R243H		R539W		R555C	
Time (s)	0	25	0	25	0	25	0	25
Mean of tail current amplitude (pA)	-406	-167	-171	-28.4	-72.8	-54.8	-110	-7.43
SEM	148	57.7	80.8	12.6	11.8	12.1	24.7	1.81
n	11	11	6	6	6	6	6	6
p value (one-tail paired t-test)	0.021		0.046		0.043		0.005	
Significant difference	YES		YES		YES		YES	

Table S2: Magnesium-induced rundown.

	WT		R243H		R539W		R555C		WT without Ci-VSP	
Time (s)	0	64	0	64	0	64	0	64	0	64
Mean of tail current density (pA/pF)	25.4	10.2	6.87	1.57	5.47	5.88	0.00	0.00	13.4	13.3
SEM	6.15	2.81	2.06	0.55	1.19	1.49	0.00	0.00	7.64	8.20
n	11	11	6	6	9	9	7	7	7	7
P value (one-tail paired t-test)	0.01		0.01		0.38		N/A		0.45	
Significant difference	YES		YES		NO		N/A		NO	

Table S3: Ci-VSP-induced rundown.
